# Cardiac mitofusin-1 is reduced in non-responding patients with idiopathic dilated cardiomyopathy

**DOI:** 10.1038/s41598-021-86209-y

**Published:** 2021-03-24

**Authors:** Yung Ting Hsiao, Ippei Shimizu, Takayuki Wakasugi, Yohko Yoshida, Ryutaro Ikegami, Yuka Hayashi, Masayoshi Suda, Goro Katsuumi, Masaaki Nakao, Takuya Ozawa, Daisuke Izumi, Takeshi Kashimura, Kazuyuki Ozaki, Tomoyoshi Soga, Tohru Minamino

**Affiliations:** 1 Department of Cardiovascular Biology and Medicine, Niigata University Graduate School of Medical and Dental Sciences, Niigata, 951-8510 Japan; 2 Japan Agency for Medical Research and Development-Core Research for Evolutionary Medical Science and Technology (AMED-CREST), Japan Agency for Medical Research and Development, Tokyo, Japan; 3 Division of Molecular Aging and Cell Biology, Niigata University Graduate School of Medical and Dental Sciences, Niigata, 951-8510 Japan; 4 Institute for Advanced Biosciences, Keio University, Yamagata, 997-0052 Japan; 5 Department of Cardiovascular Biology and Medicine, Juntendo University Graduate School of Medicine, 2-1-1 Hongo, Bunkyo-ku, Tokyo, 113-8421 Japan

**Keywords:** Medical research, Cardiovascular diseases

## Abstract

Prognosis of severe heart failure remains poor. Urgent new therapies are required. Some heart failure patients do not respond to established multidisciplinary treatment and are classified as “non-responders”. The outcome is especially poor for non-responders, and underlying mechanisms are largely unknown. Mitofusin-1 (Mfn1), a mitochondrial fusion protein, is significantly reduced in non-responding patients. This study aimed to elucidate the role of Mfn1 in the failing heart. Twenty-two idiopathic dilated cardiomyopathy (IDCM) patients who underwent endomyocardial biopsy of intraventricular septum were included. Of the 22 patients, 8 were non-responders (left ventricular (LV) ejection fraction (LVEF) of < 10% improvement at late phase follow-up). Electron microscopy (EM), quantitative PCR, and immunofluorescence studies were performed to explore the biological processes and molecules involved in failure to respond. Studies in cardiac specific Mfn1 knockout mice (c-Mfn1 KO), and in vitro studies with neonatal rat ventricular myocytes (NRVMs) were also conducted. A significant reduction in mitochondrial size in cardiomyocytes, and Mfn1, was observed in non-responders. A LV pressure overload with thoracic aortic constriction (TAC) c-Mfn1 KO mouse model was generated. Systolic function was reduced in c-Mfn1 KO mice, while mitochondria alteration in TAC c-Mfn1 KO mice increased. In vitro studies in NRVMs indicated negative regulation of Mfn1 by the β-AR/cAMP/PKA/miR-140-5p pathway resulting in significant reduction in mitochondrial respiration of NRVMs. The level of miR140-5p was increased in cardiac tissues of non-responders. Mfn1 is a biomarker of heart failure in non-responders. Therapies targeting mitochondrial dynamics and homeostasis are next generation therapy for non-responding heart failure patients.

## Introduction

Heart failure has become a global problem^1^. In addition to various medications, including: β-blockers; renin-angiotensin system modulators; nitrates; inotropic agents, and diuretics, electrical therapies such as cardiac resynchronization therapy (CRT) and mechanical support (including the left ventricular assist device: LVAD) have been developed to expand the therapeutic options for patients and have improved the outcome in some clinical settings. In particular, CRT with or without an implantable cardioverter defibrillator is reported to improve symptoms and reduce hospitalization for heart failure and overall mortality in patients with severe heart failure and intraventricular conduction delay^2^. The LVAD has also contributed to marked improvement in the quality of life of patients with severe heart failure^3^. Thus, some progress has been made in this field, however the prognosis of severe heart failure remains unacceptably poor and there is an urgent need to understand the pathology of this critical condition and discover new therapeutic targets. Even when patients receive the current optimal conventional multidisciplinary therapy, some do not show favorable improvement and have thus been described as “non-responders”. The clinical outcome is particularly poor for non-responders and the mechanisms underlying such refractory heart failure are largely unknown.

Clinical studies have identified several factors associated with improved clinical outcomes. For example, a higher systolic blood pressure (> 100 mmHg) at discharge from hospital was reported to be associated with a better prognosis^4^. In addition, some echocardiographic and electrocardiographic parameters (such as a larger ejection fraction (EF), smaller left ventricular (LV) end-diastolic diameter, and shorter QRS complex duration) predict normalization of the left ventricular ejection fraction (LVEF)^5^. Moreover, the area of late gadolinium enhancement (LGE) on cardiac magnetic resonance imaging (MRI) was reported to be a predictor of long-term outcomes^6^. Recently, elevated expression of *MYLK* (the transcript for myosin light chain kinase) and *IL6* (transcript for interleukin-6) was reported in patients responding to LVAD therapy^7^, and expression of particular circulating miRNAs was increased in responders to CRT^8^. Therefore, an important target of research is the investigation of the molecules and/or mechanisms involved in the pathology of severe heart failure, especially potential targets for new therapies.

Metabolic remodeling is well known to occur in the failing heart. Under physiological conditions, more than 95% of adenosine triphosphate (ATP) is generated from oxidative phosphorylation in the heart, mainly by utilization of fatty acids (FAs), with the remaining 5% produced by glycolysis^9^. It is generally thought that utilization of glucose increases as heart failure progresses, with concomitant reduction in the utilization of FAs^10^. In a rat model of LV pressure overload, cardiac mitochondria developed abnormal morphology and showed a reduction in density, along with a decrease of many proteins involved in the electron transport chain (ETC)^11^. Expression of genes for molecules involved in mitochondrial biogenesis, such as *Tfam* (the gene for mitochondrial transcription factor A) and *Pparα* (gene for peroxisome proliferator-activated receptor alpha), are suppressed in the infarct cardiac tissue of rats^12^. In failing human hearts, expression of *ESRRA* (gene for steroid hormone receptor estrogen receptor-like 1 [ERR1]) and *TFAM* were also reported to be reduced^13^, indicating that mitochondrial biogenesis was depressed in heart failure. It is well accepted that mitochondrial biogenesis is regulated through the processes of mitochondrial fusion and fission^14^. Molecules such as mitofusin-1 (Mfn1), mitofusin-2 (Mfn2), and dynamin-like 120 kDa protein, mitochondrial, are involved in mitochondrial fusion, while mitochondrial fission is promoted by dynamin-1-like protein (Drp1) and mitochondrial fission 1 (Fis1) protein. Papanicolaou et al. reported that cardiomyocyte-specific depletion of Mfn1 and Mfn2 leads to the onset of cardiomyopathy by postnatal day 7^15^. Inducible cardiac-specific depletion of Mfn1 and Mfn2 also showed a lethal dilated cardiomyopathy phenotype in adult mice^16^. Another group showed that cardiomyocyte-specific depletion of *Drp1* (gene for Drp1) resulted in lethal dilated cardiomyopathy and cardiomyocyte necrosis^17^. Thus, there is accumulating evidence that molecules involved in mitochondrial dynamics play a crucial role in the maintenance of mitochondrial integrity and cardiac function, however the exact role of these molecules in human heart failure remains to be determined and if there is involvement in these molecules in the pathology of non-responders remains to be explored.

In the present study, cardiac expression of Mfn1 was reduced in non-responding patients with idiopathic dilated cardiomyopathy (IDCM) at both the protein and transcriptional levels. This reduction of Mfn1 was associated with a significant decrease in both the size and number of mitochondria in cardiomyocytes. In vitro studies showed that depletion of the *Mfn1* gene led to metabolic dysfunction in neonatal rat ventricular myocytes (NRVMs), and that adrenergic receptor/cAMP/PKA/miR-140-5p pathway negatively regulated Mfn1 expression. Studies analyzing human cardiac tissues showed miR-140-5p increased in non-responding patients with heart failure. The results of the current study indicate that suppression of Mfn1 expression and disruption of mitochondrial dynamics are involved in the pathology of patients with non-responsive heart failure.

## Results

### Cardiac expression of Mfn1 is reduced in non-responders

**Figure 1:**
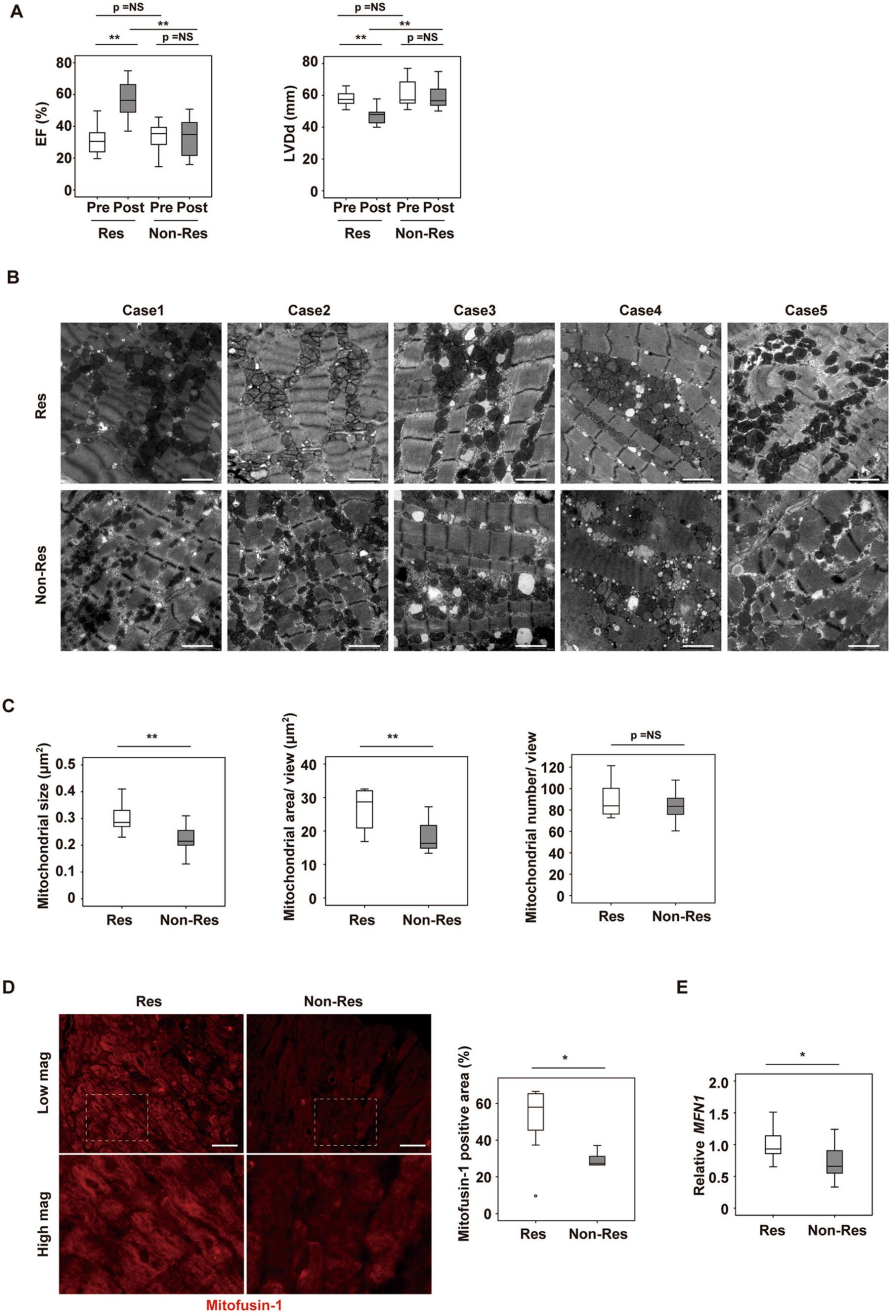
Cardiac Mfn1 expression is suppressed in non-responders together with reduction in the size and number of mitochondria in cardiomyocytes. **(A)** UCG data of responders (Res) and non-responders (Non-Res) at baseline cardiac biopsy (pre) and 7–15 months later (post). LVEF and LV diastolic dimension (LVDd) (n = 14, 14, 8, and 8). (B, C) Transmission EM of cardiac tissue from Res and Non-Res **(B)**, and quantification of the size, area and number of mitochondria per field (n = 10, 8) **(C)**. Scale bar = 2.0 μm. Case 1–5 in Fig. 1B indicates panels are from different individuals. **(D)** Immunofluorescence for Mfn1 in cardiac tissue from Res and Non-Res. Right panel shows quantification of Mfn1-positive area (n = 12, 5). Scale bar = 50 μm. For this study, an outlier (n = 1 in Res group) and an abnormal value (n = 1 in Non-Res group) were excluded by boxplot (SPSS) for further statistical analysis. **(E)** Quantitative PCR of cardiac *MFN1* expression in Res and Non-Res (n = 13, 8). For this study, an abnormal value (n = 1 in Res) was excluded by boxplot (SPSS) for further statistical analysis. Data were analyzed by two-tailed Student’s t-test. *P < 0.05, **P < 0.01. Results are shown as mean ± SEM. *NS* not significant. Small circle indicates outlier, triangle indicates abnormal value.

Patients with IDCM who underwent endomyocardial biopsy at the intraventricular septum were enrolled in this study and optimum multidisciplinary therapy was provided for all patients after biopsy. Patients were classified as non-responders when the LVEF did not show > 10% improvement on follow-up UCG performed at 7 and 15 months after biopsy (Fig. 1A). These criteria were based on a previous report that defined reverse LV remodeling as elevation of the LVEF by ≥ 10% on cardiac MRI^6^. Among the 22 patients enrolled in this study, 14 patients were classified as responders and 8 patients as non-responders. The general characteristics of the two groups were similar at hospitalization, discharge and at follow-up, except for the data of the follow-up UCGs (Tables 1, 2,3,4). Examination of the biopsy specimens by transmission EM revealed that both the size and area of the mitochondria were significantly lower in the cardiomyocytes of non-responders than in those of responders, but the number of mitochondria was similar in the groups (Fig. 1B,C). This reduction of mitochondrial size in the cardiomyocytes of non-responders promoted us to investigate changes in molecules involved in mitochondrial dynamics. It is well known that mitochondrial homeostasis is maintained through the processes of mitofusion and mitofission^18,19^. Among several candidates, immunofluorescence showed that expression of Mfn1 was significantly reduced in the cardiac tissue of non-responders (Fig. 1D, Supplemental Fig. 1A). We found a transcript for mitofusin-1 (*MFN1)* significantly reduced in failing hearts by analyzing RNA-seq data in public database (Table 5) (https://www.ncbi.nlm.nih.gov/geo/query/acc.cgi?acc=GSE116250). Expression of the transcript *MFN1* was also significantly reduced in the non-responders (Fig. 1E), while the expression profiles of the transcripts of other molecules involved in mitofusion (*MFN2* and *OPA1*), mitofission (*DNM1L* and *FIS1*), and autophagy (*BNIP3* and *MAP1LC3A*) were similar between the two groups (Supplemental Fig. 1B). We also found that the Mitofusin-2 level was similar in non-responders and responders (Supplemental Fig. 1C). The expression of mitochondrial markers (*MT-ND5*, MT-CYB, *NDUFA1*, *NDUFA9,* and *ATP5F1A*), mitochondrial DNA/ nuclear DNA ratio, metabolic markers (*CPT1A*, *CPT1B*, *PPARGC1A*, *SLC2A1* (GLUT1), *SLC2A4* (GLUT4), *HK1,* and *PKM*), fibrosis markers (*COL1A1, COL3A1* and *TGFB1*), and inflammatory markers (*CD68* and *CCL2*) were also comparable between the two groups (Supplemental Fig. 1D–G). A transcript for peroxisome proliferator-activated receptor gamma coactivator 1-alpha (PGC1α) did not differ between the groups; however, interestingly, expression of this molecule was significantly lower in the non-responding group (Supplemental Fig. 1H,I). These results suggest that, in addition to reduced PGC1α expression, cardiac mitochondrial dynamics were impaired in non-responders, particularly in the fusion process, possibly via down-regulation of Mfn1.

**Table 1:** Background of patients enrolled in this study at hospitalization.

		Responder	Non-responder	p value	n
At hospitalization	Age (years)	58.1 ± 12.1	56.5 ± 13.5	0.772	14.8
	Height (cm)	166.3 ± 9.9	164.2 ± 9.1	0.622	14.8
	Weight (kg)	72.4 ± 16.2	66.3 ± 22.0	0.462	14.8
	Heart rate (bpm)	98.7 ± 31.2	85.6 ± 28.4	0.34	14.8
	Systolic pressure (mmHg)	128.6 ± 33.3	122.4 ± 33.2	0.675	14.8
	Diastolic pressure (mmHg)	75.9 ± 26.9	76.1 ± 12.3	0.979	14.8
Echocardiogram	LVDd (mm)	58.3 ± 4.4	61.2 ± 9.0	0.415	14.8
	LVDs (mm)	49.7 ± 3.4	51.1 ± 8.9	0.693	14.8
	EF (%)	31.1 ± 8.4	33.4 ± 9.9	0.566	14.8
Catheter	LVSP (mmHg)	126.0 ± 27.1	110.0 ± 26.7	0.336	10.4
	LVEDP (mmHg)	10.0 ± 9.9	16.8 ± 7.6	0.242	11.4
	mean PAP (mmHg)	17.5 ± 7.7	18.6 ± 8.6	0.755	14.8
	mean RAP (mmHg)	3.1 ± 2.96	3.1 ± 3.6	0.974	13.8
	CI (ml/min/m^2^)	2.8 ± 0.8	2.2 ± 0.5	0.114	14.7
Lab	Cre (g/dL)	2.1 ± 2.8	0.9 ± 0.3	0.153	14.8
	Hb (g/dL)	13.7 ± 2.6	13.9 ± 1.3	0.796	14.8
	BNP (pg/ml)	599.8 ± 548.4	501.4 ± 521.9	0.686	13.8
Drugs	β blocker	3(25.0%)	6(75.0%)	0.028	12.8
	ACE-i/ARB	6(50.0%)	5(62.5%)	0.582	12.8
	Spironolactone	3(25.0%)	4(50.0%)	0.251	12.8
	Diuretics	6(50.0%)	6(75.0%)	0.264	12.8
	Amiodarone	0(0%)	0(0%)	NS	12.8

Background of responders or non-responders at hospitalization. Data were analyzed by two-tailed Student’s t-test, except for data for drugs. This data was analyzed by Chi-square test. Results are shown as mean ± SD.

*NS* not significant.

**Table 2:** Medication of patients enrolled in this study at discharge.

At discharge		Responder	Non-responder	p value	N
Drugs	β blocker	13 (92.9%)	8 (100%)	0.439	14.8
	ACE-i/ARB	12 (85.7%)	7 (87.5%)	0.907	14.8
	spironolactone	11 (78.6%)	6 (75.0%)	0.848	14.8
	Diuretics	11 (78.6%)	7 (87.5%)	0.601	14.8
	amiodarone	0( 0%)	0 (0%)	NS	14.8

Drugs of responders or non-responders at discharge. Data were analyzed by Chi-square test. Results are shown as mean ± SD.

*NS* not significant.

**Table 3:** Background of patients enrolled in this study at follow up.

At follow up		Responder	Non-responder	p value	N
Echocardiogram	LVDd (mm)	49.0 ± 7.7	59.3 ± 8.3	0.008	14.8
	LVDs (mm)	34.6 ± 8.5	50.6 ± 10.3	0.001	14.8
	EF (%)	57.2 ± 11.5	33.1 ± 12.9	< 0.001	14.8
Lab	Cre (g/dL)	2.6 ± 4.2	1.2 ± 0.5	0.409	13.6
	Hb (g/dL)	13.4 ± 2.3	15.1 ± 1.6	0.115	13.6
	BNP (pg/ml)	150.3 ± 175.2	96.6 ± 73.2	0.494	9.6
Drugs	β blocker	12 (100%)	8 (100%)	NS	12.8
	ACE-i/ARB	9 (75%)	8 (100%)	0.125	12.8
	spironolactone	10 (83.3%)	6 (75.0%)	0.648	12.8
	Diuretics	9 (75%)	7 (87.5%)	0.494	12.8
	amiodarone	0 (0%)	1 (12.5%)	0.191	12.8

Background of responders or non-responders at follow-up. Data were analyzed by two-tailed Student’s t-test, except for data for drugs. This data was analyzed by Chi-square test. Results are shown as mean ± SD.

*NS* not significant.

**Table 4:** Diagnosis, comorbidity and other patient characteristics enrolled in this study.

	Diagnosis	Comorbidity	Gender	Group	Age	Height (cm)	Weight (kg)
Case 1	DCM	DL	M	Responder	74	157	70.1
Case 2	DCM	HUA, SAS	M	Responder	52	158	65
Case 3	DCM	HTN, CRF on HD, DM	M	Responder	37	184.7	95.2
Case 4	DCM	Liver cirrhosis, HCV, Type2 DM, HTN	M	Responder	64	171	79.4
Case 5	DCM	Afib, Type2 DM, HTN	M	Responder	68	177	88.3
Case 6	DCM	Spinal canal stenosis	M	Responder	61	161	63.9
Case 7	DCM	Afib, bronchial asthma, SAS	M	Responder	63	175	86
Case 8	DCM	HTN	M	Responder	45	179	94
Case 9	DCM	AS, COPD, DL	M	Responder	76	154	56
Case 10	DCM	CRF on HD, DL, HTN, ANCA glomerulonephritis	M	Responder	56	157.7	51.9
Case 11	DCM	HTN, Type2 DM, bronchial asthma, HUA	M	Responder	53	164	88.3
Case 12	DCM	Bronchial asthma, DM, adipoma, DL	F	Responder	50	158.1	57.5
Case 13	DCM	Bronchial asthma, Afib	M	Responder	43	172	71.2
Case 14	DCM	Afib	M	Responder	72	160	46.8
Case 15	DCM	N/A	M	Non-responder	37	166.4	61.6
Case 16	DCM	HUA、SAS	M	Non-responder	39	169.2	62
Case 17	DCM	HTN, DL, DM, AAA post grafting, Afib, DM nephropathy	M	Non-responder	63	169	83.3
Case 18	DCM	Type2 DM	M	Non-responder	50	168.8	100.1
Case 19	DCM	nsVT, Afib	F	Non-responder	75	142.1	31.2
Case 20	DCM	HTN, Type2 DM	M	Non-responder	65	164	50.4
Case 21	DCM	Afib, Type2 DM, DL, HTN, CKD, SAS	M	Non-responder	66	168	84.6
Case 22	DCM	Afib, fatty liver	M	Non-responder	57	166	57

DL (dyslipidemia), HUA (hyperuricemia), SAS (sleep apnea syndrome), HTN (hypertension), CRF (chronic renal failure), HD (hemodialysis) DM (diabetes mellitus), HCV (hepatitis C virus), Afib (atrial fibrillation), AS (aortic stenosis), COPD (chronic obstructive pulmonary disease) AAA (abdominal aorta aneurysm), nsVT (non-sustained ventricular tachycardia).

**Table 5:** RNA seq study analyzing transcripts MFN1 or NPPB (transcript for BNP) in public database.

Series ID	Method	Gene	DCM/Con ratio	p value	Sample case number	Source
GSE 116250	RNA-seq	MFN1	0.8149743	0.01419	Con n = 14 DCM n = 37	https://www.ncbi.nlm.nih.gov/geo/query/acc.cgi?acc=GSE116250
		NPPB	13.9046589	0.00118	Con n = 14 DCM n = 37	

### Suppression of Mfn1 promotes metabolic dysfunction in cardiomyocytes

**Figure 2:**
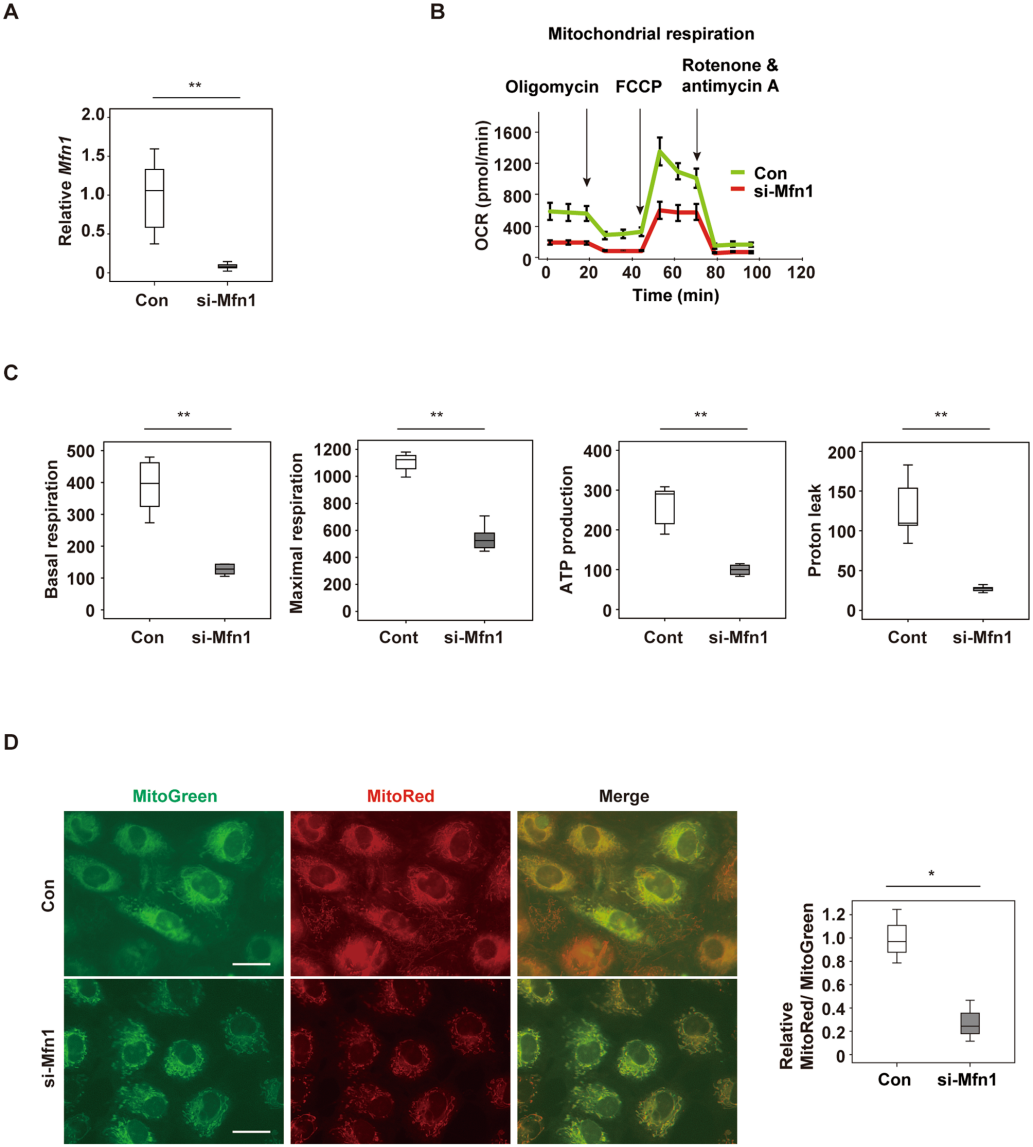
Suppression of Mfn1 promotes the decline of cardiomyocytes metabolism **(A)** Quantitative PCR of *Mfn1* expression in NRVMs after introduction of control si-RNA (Con) or si-Mfn1 (n = 6, 6). **(B,C)** Evaluation of mitochondrial respiration with the Seahorse extracellular flux analyzer **(B)**, basal respiration (n = 5, 5), maximal respiration (n = 4, 5), ATP production (n = 5, 5), and Proton leak (n = 5, 5). **(C)** In NRVMs after introduction of control si-RNA (Con) or si-Mfn1. For maximal respiration study, an abnormal value (n = 1 in si-Mfn1 group) was excluded by boxplot (SPSS) for further statistical analysis. **(D)** Staining of functional mitochondria (MitoRed) and total mitochondria (Mito Tracker Green FM (MitoGreen)) in NRVMs after introduction of control si-RNA (Con) or si-Mfn1. Scale bar = 5 μm. The right panel shows quantification of the MitoRed-positive area (n = 3, 3). Data were analyzed by two-tailed Student’s t-test. *P < 0.05, **P < 0.01. Results are shown as mean ± SEM. *NS* not significant. Small circle indicates outlier, triangle indicates abnormal value.

To investigate the role of Mfn1 in cardiac tissue, specific depletion of the gene for this molecule, *MFN1* in NRVMs was induced by introduction of si-RNA, si-*Mfn1*. Introduction of si-*Mfn1* significantly reduced *Mfn1* expression in NRVMs (Fig. 2A). Extracellular flux analysis showed that depletion of *Mfn1* significantly reduced mitochondrial respiration in NRVMs (Fig. 2B,C), along with a significant decrease of the mitochondrial membrane potential (Fig. 2D). The expression of transcripts for ATP synthase subunit alpha, mitochondrial (*Atp5f1a1*), NADH-ubiquinone oxidoreductase chain 5 (*Mtnd5*) and Ndufa1 protein (*Ndufa1*) were unchanged by depletion of *Mfn1* (Supplemental Fig. 2A). There was a trend in a reduction of glycolytic function in response to *Mfn1* suppression (Supplemental Fig. 2B,C). Transcripts of rate-limiting enzymes for glycolysis including *Hk1* (hexokinase-1), *Pfkm* (ATP-dependent 6-phosphofructokinase) and *Pkm* (pyruvate kinase PKM) were comparable before and after introduction of si-*Mfn1* (Supplemental Fig. 2D). Taken together, these data indicate that down-regulation of *Mfn1* promotes metabolic remodeling in cardiomyocytes primarily through suppression of mitochondrial respiration. Production of ATP is highly dependent on oxidative phosphorylation in the mitochondria^20^. Cardiac levels of ATP and phosphocreatine decrease with the progression of heart failure, and the heart “runs out of fuel” in the advanced stage of this fatal condition^21^. The results of the present study suggest that suppression of Mfn1 promotes metabolic remodeling in cardiac tissues and contributes to the failing heart.

### Cardiac Mfn1 KO mice show reduced cardiac function

**Figure 3:**
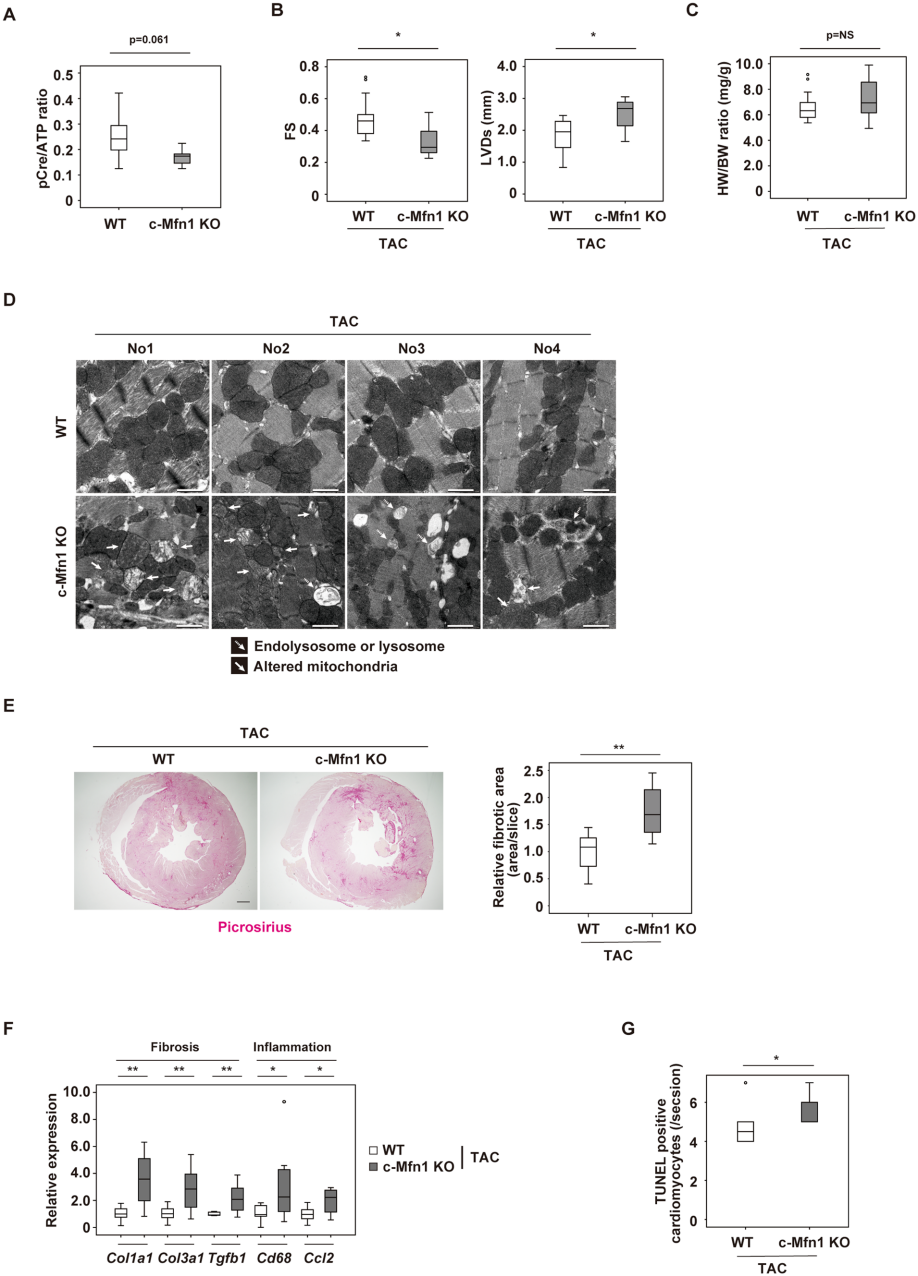
Cardiac Mfn1 KO mice show reduced cardiac function. **(A)** Metabolomic study showing pCre/ATP ratio in cardiac tissues of wild type (WT) or MHC-Cre^+/−^; Mitofusin-1^fl/fl^ (c-Mfn1 KO) mice (n = 7, 8). An abnormal value (n = 1 in the c-Mfn1 KO group) was excluded by boxplot (SPSS) for further statistical analysis. **(B)** echocardiography data of WT and c-Mfn1 KO mice two weeks after TAC. Fractional shortening (FS) (n = 18, 10) and LV systolic dimension (LVDs) (n = 18, 10) were analyzed. **(C)** Heart weight/body weight (HW/BW) ratio of indicated groups (n = 18, 10). **(D)** Transmission EM of cardiac tissues from WT and c-Mfn1 KO mice two weeks after TAC. No 1–4 indicates panels from different mice. Scale bar = 1 μm **(E)** Picrosirius staining of indicated mice. Right panel indicates relative fibrotic area (area/slice) (n = 5, 6). An abnormal value (n = 1 in c-Mfn1 KO) was excluded by boxplot (SPSS) for further statistical analysis. Scale bar = 500 μm. **(F)** Quantitative PCR of *Col1a1* (n = 10, 11), *Col3a1* (n = 10, 11), *Tgfb1* (n = 8, 11), *Cd68* (n = 8, 10) and *Ccl2* (n = 10, 11) in cardiac tissues of indicated mice. An outlier (n = 1 in c-Mfn1 KO (*Cd68*)) and an abnormal value (n = 1 in WT (*Tgfb1)*) were excluded by boxplot (SPSS) for further statistical analysis. In WT (*Tgfb1*), n = 1, WT (*Cd68*), n = 2, were not detected, and also excluded from the analyses. **(G)** TUNEL positive cardiomyocytes in the indicated group (n = 5, 7). An outlier (n = 1 in WT) was excluded by boxplot (SPSS) for further statistical analysis. Data were analyzed by two-tailed Student’s t-test. *P < 0.05, **P < 0.01. Results are shown as mean ± SEM. NS = not significant. Small circle indicates outlier, triangle indicates abnormal value.

To further test the effect of Mfn1 suppression in in vivo settings, a cardiac specific Mfn1 knockout mouse model was generated by crossing MHC-Cre mice with Mfn1^fl/fl^ mice resulting in c-Mfn1 KO. It was confirmed that there was a significant reduction in *Mfn1*, but not *Mfn2, Opa1, Dnm1l, Fis1* expression, in cardiac tissues of c-Mfn1 KO mice (Supplemental Fig. 3A). At baseline, UCG findings indicated that cardiac function of c-Mfn1 KO mice was comparable with that of littermate controls; furthermore, c-Mfn1 KO exhibited mild LV hypertrophy compared with the wild-type mice (Supplemental Fig. 3B). This finding is similar to a previous report by Papanicolaou et al.^22^. A reduction in mitochondrial size in the hearts of c-Mfn1 KO mice was observed via EM, and metabolomic studies showed reduced phospho-creatine/ATP ratio in cardiac tissues of c-Mfn1 KO mice (Fig. 3A, Supplemental Fig. 3C,D). Several other metabolites were shown to either increase (G6P, F6P, F1,6P, AcetylCoA, NADH, N6N6N6-Trimethyllysine, Cytidine, and Clycerophosphorylcholine) or decrease (Cardnosine, Pro, Citrulline, His and Ser) in these mice (Supplemental Fig. 3E,F). A LV pressure overload model with TAC was then generated. During LV pressure overload, c-Mfn1 KO mice showed reduced systolic function with enhanced cardiac dilatation (Fig. 3B, Table 6). Heart weight/body weight ratio was comparable between the TAC and the non-TAC groups (Fig. 3C). An increase of lysosome or mitochondrial alteration in cardiomyocytes was observed via EM (Fig. 3D). Fibrotic area increased in c-Mfn1 KO mice during LV pressure overload along with an increase in transcripts for fibrotic and inflammatory markers (Fig. 3E,F). TUNEL positive cardiomyocytes increased in c-Mfn1 KO mice compared with WT mice after TAC operation, suggesting that apoptotic cardiomyocytes promote replacement fibrosis in the c-Mfn1 KO that underwent the TAC operation group (Fig. 3G).

**Table 6:** Echocardiography data.

Parameters (mean ± SD)	WT (n = 18)	c-Mfn1 KO (n = 10)	*P*
FS (%)	0.48 ± 0.03	0.33 ± 0.03	0.025
LVDs (mm)	1.82 ± 0.13	2.51 ± 0.16	0.021
IVSTd (mm)	1.10 ± 0.03	1.11 ± 0.02	0.116
HR (bpm)	631.22 ± 11.80	645.60 ± 20.16	0.638

Each cardiac parameter (mean ± SE) measured by echocardiography. Wild-type (WT) and cardiac-specific Mfn1 knock-out (c-Mfn1 KO) mice were observed after 2 weeks of TAC operation. Data were analyzed by two-tailed Student’s t-test. Results are shown as mean ± SD.

### Continuous adrenergic signaling reduces Mfn1 expression

**Figure 4:**
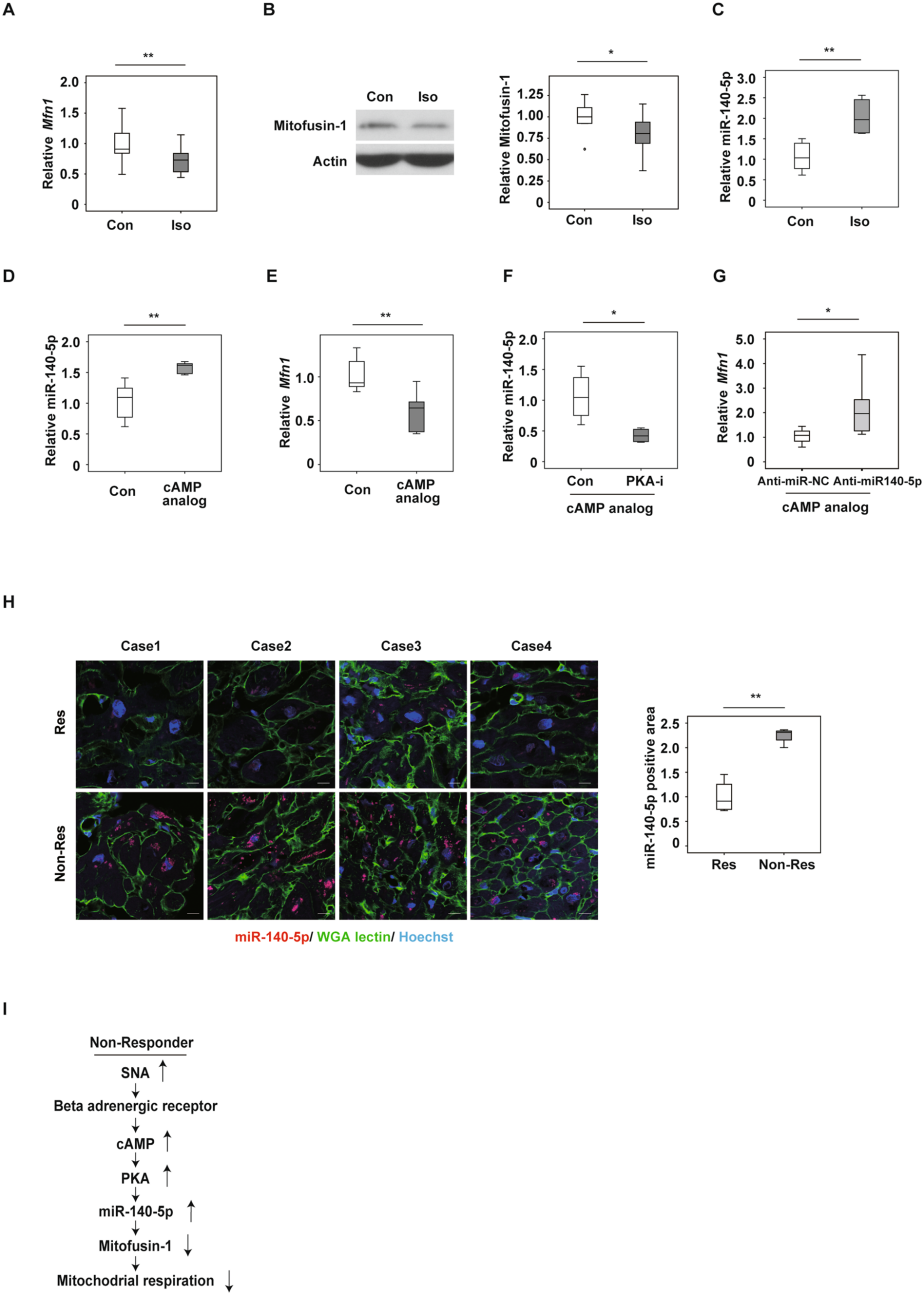
Activation of adrenergic signaling reduces mitofusin-1 expression. **(A,E)** Quantitative PCR of *Mfn1* in NRVMs treated with PBS (Con) or isoproterenol (Iso, 1 μM, 72 h) (n = 18, 14) **(A)**, PBS (Con) or cAMP analog (30 μM, 24 h) (n = 6,6) **(E)**.** (B)** Western blot analysis of Mfn1 in NRVMs administrated with PBS (Con) or isoproterenol (Iso, 1 μM, 96 h). **(B)** Right panel, quantification for Mfn1 relative to Actin loading control (n = 8, 9). **(C,D,F)** Quantitative PCR of miR-140-5p in NRVMs administrated with isoproterenol (Iso, 1 μM, 72 h). **(C)** cAMP analog (30 μM, 3 h, n = 6) **(D)** cAMP analog (30 μM, 3 h) + PKA inhibitor (PKA-I, 2 μM, 30 min pre-treatment) (n = 6, 6) **(F)** (n = 4, 4). **(G)** Quantitative PCR of *Mfn1* in NRVMs subjected to cAMP analog (30 μM, 24 h) + anti-miR-140-5p (50 nM pretreatment, 1 h) (n = 9, 9) or anti-miR negative control (Anti-miR-NC). **(H)** In situ hybridization detecting miR-140-5p in cardiac tissue of responders (Res) (n = 4) or non-responders (Non-Res) (n = 4). Right panel indicates miR-140-5p positive (n = 4, 4) (scale bar = 10 μM). **(I)** Scheme summarizing findings of β-AR/ cAMP/ PKA/ miR-140-5p signaling pathways leading to down-regulation of Mfn1 expression in cardiac tissues of non-responding patients with heart failure. Data were analyzed by two-tailed Student’s t-test. *P < 0.05, **P < 0.01. Results are shown as mean ± SEM. *NS* not significant. Small circle indicates outlier, triangle indicates abnormal value.

It has been reported that heart failure patients have high circulating catecholamine levels, which are linked with poor clinical outcomes^23^. Activation of adrenergic signaling promotes pathologies in the failing heart, and β-blockers are well recognized as a first line therapeutic option for patients with heart failure. The β-adrenergic receptor (β-AR)/ cAMP/ PKA/ signaling pathway is one of the most well-characterized pathways, and downstream molecules, including CaMKII, have been reported to introduce pathological cardiac hypertrophy, associated with reduced cardiac function^24^. To further assess the role of adrenergic signaling in the regulation of Mfn1 expression, NRVMs were incubated in the presence of isoproterenol, a β-AR agonist. Isoproterenol treatment led to a significant reduction in Mfn1 expression (Fig. 4A,B), and this was inhibited by incubation with a PKA inhibitor (Supplemental Fig. 4A). MicroRNA (miRNA) contributes to the post-transcriptional regulation of target mRNAs, and studies have indicated the pivotal role of miRNA in cardiac tissues under physiological or pathological conditions^25^. An in-silico study analyzing Target Scan (http://www.targetscan.org/vert_72/) indicated several miRNAs that may have roles in the regulation of *Mfn1* mRNA. From the findings of this previous study, the present investigation focused on miR-140-5p, as miR-140-5p was previously reported to suppress *Mfn1* in cardiomyocytes^26^. In NRVMs, isoproterenol increased miR-140-5p expression (Fig. 4C). Administration of a cAMP analog also increased miR-140-5p expression (Fig. 4D), and under these conditions, *Mfn1* expression was suppressed (Fig. 4E). A PKA inhibitor suppressed miR-140-5p expression (Fig. 4F), and inhibition of this miRNA with anti-miR140-5p led to an increase in *Mfn1* expression (Fig. 4G). Finally, it was observed that cardiac tissues from non-responders exhibited high expression profiles for miR140-5p (Fig. 4H). Taken together, these results suggest that the β-AR/cAMP/PKA/miR-140-5p signaling pathway down-regulates Mfn1 expression in cardiac tissues of non-responding patients with heart failure (Fig. 4I). Developing methods for activation of Mfn1 and mitochondrial dynamics could lead to next generation therapy for severe heart failure.

## Discussion

Heart failure has a complex pathogenesis, and current simple therapeutic approaches are insufficient to manage this difficult condition. Both β-blockers and renin-angiotensin system modulators are currently first-line therapy for patients with heart failure with a reduced ejection fraction (HFrEF). Heart failure is treated with a combination of several drugs plus electrical and mechanical supports such as CRT or LVAD, depending on the stage of the illness (reviewed by Udelson et al.^25^ and Abraham et al.^26^). However, not all patients show a favorable response to clinically optimum therapy. The prognosis of non-responders is poor^27^ and the mechanisms of underlying refractoriness currently remain elusive, possibly due to the lack of animal and in vitro models for this condition. In the present study, in the non-responding patients with IDCM, EM showed that the cardiomyocytes had significantly smaller mitochondria together with reduced expression of Mfn1. Mitochondrial homeostasis is maintained by the twin processes of fusion and fission, and previous studies have indicated that disruption of mitochondrial dynamics leads to cardiac dysfunction in rodents^15,17^. It has been reported that many of the molecules involved in mitochondrial biogenesis show reduced expression in the failing hearts of rodents and humans^11,13,28–30^. Interestingly, the present study showed that the cardiac tissue level of expression of transcripts for various molecules involved in mitofusion (*MFN2* and *OPA1*), mitofission (*DNM1L* and *FIS1*), and autophagy (*BNIP3* and *MAP1LC3A*) were similar between responders and non-responders. In vitro experiments indicated that depletion of *Mfn1* with si-RNA promoted metabolic dysfunction, characterized by reduced mitochondrial respiration, in NRVMs. Impairment of metabolism has been reported to occur with the progression of heart failure, and the findings of the present study indicate that Mfn1 may be critically involved in this detrimental process.

Heart failure patients have high blood catecholamine levels, and it is well known that increased adrenergic tone is correlated with a poor prognosis^2^. The in vitro studies using NRVMs indicated that exposure to isoproterenol reduced the expression of Mfn1, and this was ameliorated with a PKA inhibitor. In silico analysis confirmed the in vitro studies, and indicated that the β-AR/cAMP/PKA/miR-140-5p signaling pathway suppresses *Mfn1* mRNA, however the underlying mechanisms contributing to the increase in miR-140-5p remains to be explored. A high affinity β_1_-AR blocker, metoprolol, has been reported to be beneficial for IDCM^31^. This beneficial attribute may be the ability to suppress the β_1_-AR/ cAMP/ PKA/ miR-140-5p signaling pathway, thus inhibiting the decrease of Mfn1 in failing hearts. The present study only includes a small number of patients with IDCM, and there are a number of limitations. Further studies are required to determine whether clinically beneficial drugs are effective in activating or increasing cardiac Mfn1.

## Methods

### Patients

This study was approved by the Research Ethnic Committee of Niigata University (approval number 2439). Twenty-two patients with IDCM who underwent endomyocardial biopsy of the intraventricular septum between 2012 and 2017 were retrospectively enrolled in this study. All participants gave written informed consent prior to endomyocardial biopsy. Following endomyocardial biopsy, optimal multidisciplinary therapy was provided to all patients according to the guidelines of the Japanese Circulation Society, and the study was performed in accordance with the Helsinki declaration. In addition to being conducted for initial patient characterization, follow-up echocardiography was performed at 7 and 15 months after biopsy. Patients were classified as non-responders if the LVEF did not show > 10% improvement on the follow-up echocardiograms^6^. Total RNA was extracted from the cardiac biopsy specimens of all patients for further studies. In some patients, electron microscopy (EM) was performed in addition to examination of tissue sections.

### Cell culture

The NRVMs were prepared from the hearts of 2- to 3-day-old Wistar rats. Hearts were removed, washed in ice-cold phosphate-buffered saline (PBS), minced into pieces of approximately 1 mm^3^, and washed again with cold PBS. Isolation of cells was achieved by multiple sessions of digestion using 0.05% trypsin-ethylenediaminetetraacetic acid.

(EDTA) solution at 37 °C for 6 min. After each digestion procedure, the cell suspension was immediately placed in Dulbecco’s modified Eagle’s medium (DMEM) with 10% fetal bovine serum (FBS) and 1% penicillin/streptomycin (P/S) (culture medium). After all digestions, the pooled cell suspensions in DMEM were centrifuged for 5 min at 1,000 rpm, after which the pellet was re-suspended in culture medium (DMEM with 10% FBS and 1% P/S). The re-suspended cells were passed through a 70 μm nylon filter and pre-plated on 10 cm^2^ plates for 60 min to remove fibroblasts. Unattached cells (enriched cardiomyocytes) were then plated into primary cell culture dishes. The cells were incubated at 37 °C under 5% CO_2_ conditions and the medium changed after 24 h. All animal study protocols were approved by the Niigata University review board.

### Histology

A portion of the biopsy specimen from the intraventricular septum of each IDCM patient was fixed in 10% formalin and embedded in paraffin wax. Paraffin sections were prepared at the Pathology Department of Niigata University Medical & Dental Hospital. Protein levels of the molecules of interest were evaluated by staining sections with anti-Mitofusin-1 antibody (Abcam, ab104274), Mitofusin-2 (D2D10) Rabbit mAb (Cell signaling, #9482), Anti-PGC1 alpha Ab (abcam, ab54481). The secondary antibody for these antibodies was Cy5 Goat Anti-Rabbit IgG (H + L) (Thermo Fisher Scientific, A10523). Three fields were randomly selected from each section for quantification. A portion of the biopsy specimen from several patients, was fixed in 2.5% glutaraldehyde solution and EM grids prepared by Bio Medical Laboratories, Inc., after which transmission EM was performed using a JEM1400 microscope at Niigata University Medical Campus. In each sample, we randomly selected 5 views and counted and measured the mitochondria in each view. Cardiac tissues from mice that had undergone thoracic aortic constriction (TAC) were fixed in 10% formalin, embedded in paraffin, and sectioned for Harris hematoxylin–eosin (H&E), Picrosirius Red or TdT-mediated dUTP nick end labeling (TUNEL) staining. The TUNEL labeling was performed according to the manufacturer’s protocol (In Situ Cell Death Detection Kit, Fluorescein; Roche, 1684795). Picrosirius Red staining was performed using Picrosirius Red staining Kit (COSMO BIO, 24901-250), according the manufacturer’s protocol.

### Western blot analysis

Whole-cell lysates were prepared in lysis buffer (10 mM Tris-hydrochloride [HCl], pH 8, 140 mM sodium chloride [NaCl], 10 mM EDTA, 0.025% sodium azide [NaN3], 1% Triton X-100, 1% deoxycholate, 0.1% sodium dodecyl sulphate [SDS], and 4% protease inhibitor). The lysates (20–30 μg) were then resolved by the SDS–polyacrylamide gel electrophoresis (PAGE) method and proteins transferred to a polyvinylidene fluoride (PVDF) membrane (Millipore). The PVDF membrane was then incubated with the primary antibody and then with horseradish peroxidase-conjugated anti-rabbit IgG (Jackson Laboratories). The primary antibodies used for western blotting were anti-Mitofusin 1 antibody (Abcam, ab104274) and anti-PAN-actin antibody (Cell Signaling, 4968 s), both at 1:1000, while the secondary antibody was Peroxidase AffiniPure Goat Anti-Rabbit IgG (H + L) (Jackson Immunoresearch, 111-035-003) at 1:5000.

### RNA analysis

Total RNA (1 μg) was isolated from cell samples with RNA-Bee (TEL-TEST Inc.). Quantitative polymerase chain reaction (PCR) was performed by using a Light Cycler 480 (Roche) with the TaqMan Universal Probe Library and the Light Cycler 480 Probes Master (Roche) according to the manufacturer’s instructions. The primers and their sequences were as follows:

### Human primers

*MFN1*; 5′-GGGTGCTCCTAGGATTATCAGA, 5′-TATCTGGCGTTGCTGGAGT-3′,

*MFN2*; 5′-TGCCTCAGAGCCCGAGTA-3′, 5′-CTGGTACAACGCTCCATGTG-3′,

*OPA1*; 5′-AACCATATTCGTTTTGACCAAAGT-3′, 5′-TTGGGAAGAGCTTTCCTTCA-3′,

*DNM1L*; 5′-CCGTTAGACAGTGCACCAAG-3′, 5′-GCTCCATCTCCTCCCGTAG-3′,

*FIS1*; 5′-CTGAACGAGCTGGTGTCTGT-3′, 5′-GAGCCTGCTGCCTTCTCA-3′,

*BNIP3*; 5′-GAATTTCTGAAAGTTTTCCTTCCA-3′, 5′-TTGTCAGACGCCTTCCAATA-3′,

*MAP1LC3A*; 5′-CATGAGCGAGTTGGTCAAGA-3′, 5′-CCATGCTGTGCTGGTTCA-3′,

*MT-ND5*; 5′-AAATCCATTGTCGCATCCA-3′, 5′-TTGGTCTAGGCACATGAATATTGT-3′,

*NDUFA1*; 5′-GAGAAAGCTGCTCTGCCATT-3′, 5′-GCAACGTGGAGTGACGTG-3′,

*NDUFA9*;5′-GTCCTATACCCCTTGGTTCCTT-3′, 5′-AATTCCTTTGGATACATCTACGACA-3′,

*ATP5F1A*; 5′-GATAGAGCCGCCTAGAACCA-3′, 5′-TACTCCGCAGGCGGTACTT-3′,

*CPT1A*; 5′-CAATCGGACTCTGGAAACG-3′, 5′-CCGCTGACCACGTTCTTC-3′,

*CPT1B*; 5′-GAGCAGCACCCCAATCAC-3′, 5′-AACTCCATAGCCATCATCTGCT-3′,

*PPARGC1A*; 5′-TGAGAGGGCCAAGCAAAG-3′, 5′-ATAAATCACACGGCGCTCTT-3′,

*SLC2A1*; 5′-GGTTGTGCCATACTCATGACC-3′, CAGATAGGACATCCAGGGTAGC-3′,

*SLC2A4*; 5′-CTGTGCCATCCTGATGACTG-3′, 5′-CGTAGCTCATGGCTGGAACT-3′,

*HK1*; 5′-GACACCCCAGAGAACATCGT-3′, 5′-GCACTCAGCAACATGATCAAA-3′,

*PKM*; 5′-CCTCAGCCTCACGAGCTATC-3′, 5′-CAGCCAAAGGGGACTATCCT-3′,

*COL1A1*; 5′-GGGATTCCCTGGACCTAAAG-3′, 5′-GGAACACCTCGCTCTCCA-3′,

*COL3A1*; 5′-CTGGACCCCAGGGTCTTC-3′, 5′-GACCATCTGATCCAGGGTTTC -3′,

*TGFB1*; 5′-ACTACTACGCCAAGGAGGTCAC-3′, 5′-TGCTTGAACTTGTCATAGATTTCG-3′,

*CD68*; 5′-GTCCACCTCGACCTGCTCT -3′, 5′-CACTGGGGCAGGAGAAACT -3′,

*CCL2*; 5′-AGTCTCTGCCGCCCTTCT-3′, 5′-GTGACTGGGGCATTGATTG-3′,

*MT-CYB*; 5′-CAATGGCGCCTCAATATTCT-3′, 5′-GCCGATGTTTCAGGTTTCTG-3′,

*MT-COX1*; 5′-AGCTCTAAGCCTCCTTATTCGAG-3′, 5′-CGTTGTAGATGTGGTCGTTACC-3′,

*COX6A1*; 5′-TTGGTGTGTCCTCGGTTTC-3′, 5′-GTCTTCCACATGCGAGCTG-3’.

### Rat primers

*Mfn1*; 5′-CAAACTGCAGCCACCAAGT-3′, 5′-GTTGGCACAGTCGAGCAA-3′,

*Atp5f1a1*; 5′-TGCATGGACTGAGGAACG-3′, 5′-CCAAGTTCAGGGACATACCC-3′,

*Ppargc1a*; 5′-GCAGTCGCAACATGCTCA-3′, 5′-GGGTCATTTGGTGACTCTGG-3′,

*Mtnd5*; 5′-CCAACAATCTAGTCCCTCTCACA-3′, 5′-TCATGGATGAAGTCCGAATTG-3′,

*Ndufa1*; 5′-AGACGCATCTCTGGTGTCAA-3′, 5′-GCCAGGAAAATGCTTCCTTA-3′,

*Hk1*; 5′-TCTGGGCTTCACCTTCTCAT-3′, 5′-ATCAAGATTCCACAGTCCAGGT-3′,

*Pkm*; 5′-AACAGCCAAAGGGGACTACC-3′, 5′-CCTCAGCCTCTCGAGCTATC-3′,

*Rplp0*; 5′-GATGCCCAGGGAAGACAG-3′, 5′-CACAATGAAGCATTTTGGGTAG-3′,

*Opa1*; 5′-ACCAGGAGAAGTAGACGGTGTC-3′, 5′-TTCTTCAAATGTATGCAGAGGTG-3′,

*Dnm1l*; 5′-GCTGGTCCACGTTTCACC-3′, 5′-CCCCATTCTTCTGCTTCAAC-3′,

*Fis1*; 5′- GATCATCCTCGGATGTAGGG-3′, 5′-GACTGAAATTTCCTTTCAAAATTCC-3’.

### Mouse primers

*Mfn1*; 5′-GTGAGCTTCACCAGTGCAAA-3′, 5′-CACAGTCGAGCAAAAGTAGTGG-3’,

*Mfn2*; 5′- CGAGGCTCTGGATTCACTTC -3′, 5′- CAACCAGCCAGCTTTATTCC-3’,

*Col1a1*; 5′- CATGTTCAGCTTTGTGGACCT-3′, 5′- GCAGCTGACTTCAGGGATGT -3’,

*Col3a1*; 5′- TCCCCTGGAATCTGTGAATC -3′, 5′- TGAGTCGAATTGGGGAGAAT-3’,

*Tgfb1*; 5′- TGGAGCAACATGTGGAACTC-3′, 5′- CAGCAGCCGGTTACCAAG-3’,

*CD68*; 5′- GACCTACATCAGAGCCCGAGT-3′, 5′- CGCCATGAATGTCCACTG-3’,

*Ccl2*; 5′- CATCCACGTGTTGGCTCA-3′, 5′- GATCATCTTGCTGGTGAATGAGT-3’,

*Rplp0*; 5′-GATGCCCAGGGAAGACAG-3′, 5′-ACAATGAAGCATTTTGGATAA-3’.

*Rplp0* (rat and mouse) or *COX6A1* (human) was used as the internal control for all studies.

### Extracellular flux assay

The cellular oxygen consumption rate and extracellular acidification rate were measured with a Seahorse XF extracellular flux analyzer as previously described^32^ according to the manufacturer’s instruction (Seahorse Bioscience Inc.). The NRVMs were seeded in a Seahorse XF 24-well assay plate in DMEM with 10% FBS at a density of 10,000 to 50,000 cells per well, and treated with siRNA for *Mfn1* (si-*Mfn1*; Qiagen) and negative control siRNA (Qiagen). After overnight attachment, the plate was washed and the medium replaced with pre-warmed running medium (XF base medium supplemented with 10% D-glucose, 100 mM pyruvate and 200 mM glutamine for the mitochondrial stress test, or with only 200 mM glutamine for the glycolysis stress test), followed by incubation in a non-CO_2_ incubator at 37 °C for 60 min. The basal oxygen consumption rate and extracellular acidification rate were recorded for 24 min, followed by performance of the mitochondrial stress test (1 μM oligomycin, 2 μM FCCP, and 0.5 μM rotenone/antimycin A) and the glycolysis stress test (10 mM glucose, 1 μM oligomycin, and 50 mM 2-deoxy-d-glucose [2-DG]). All reagents were from the Seahorse XF Cell Mito Stress Test Kit (Seahorse Bioscience, #103015-100) and the Seahorse XF Glycolysis Stress Test Kit (Seahorse Bioscience, #103020-100).

### Chemicals and drugs

The following drugs were used for in vitro experiments: Isoproterenol (1 μM for 72–96 h, depending on the assay; Sigma-Aldrich, #I6504), 8-bromoadenosine 3′,5′-cyclic monophosphate (30 μM, Sigma-Aldrich, #B5386) as a cAMP analog, and H89 dihydrochloride (1 μM, Tocris, #2910) as a PKA inhibitor.

### RNA interference

Small interfering RNA (siRNA) targeting *Mfn1* [a mixture of si-*Mfn1* #101907367 (25 pM) + si-*Mfn1* #101907374 (25 pM)] and the corresponding negative control (#1027280) were purchased from Qiagen. These siRNAs were transfected using Lipofectamine RNAi MAX (Invitrogen, #13778-150) and Opti-MEM (Gibco by Life Technologies, #31985-062). The medium was replaced at 24 h after addition of siRNAs, and the cells were incubated for a further 48 h before further studies were performed, unless otherwise stated.

### miRNA studies

From NRVMs, miRNA was extracted using the mirVana miRNA Isolation Kit according to the manufacturer’s instruction (Invitrogen, #AM1560). Purified miRNA was subjected to reverse transcription by TaqMan MicroRNA Reverse Transcription Kit (#4366596) and TaqMan microRNA Assay Kit (#4427975) with specific primers (mmu-miR-140 # 001187; U6 snRNA # 001973) purchased from Applied Biosystems. Expression of miR-140-5p was analyzed by a Light Cycler 480 (Roche) with TaqMan microRNA Assay Kit and TaqMan Fast Advanced Master Mix (Thermo Fisher Scientific, #4444556) according to the manufacturer’s instructions. Results from quantitative PCR were normalized with U6 snRNA. Primers and their sequences were as follows:

U6 snRNA: GTGCTCGCTTCGGCAGCACATATACTAAAATTGGAACGATACAGAGAAGATTAGC- ATGGCCCCTGCGCAAGGATGACACGCAAATTCGTGAAGCGTTCCATATTTT

mmu-miR-140: CAGUGGUUUUACCCUAUGGUAG.

Ambion Anti-miR (#AM17000) was used as a miRNA inhibitor targeting miR-140-5p (#AM10205), and the corresponding negative control (#AM17010) was also purchased from Ambion. These Anti-miRs were transfected with Lipofectamine RNAi MAX (Invitrogen, #13778-150) and Opti-MEM (Gibco by Life Technologies, #31985-062). Anti-miRs were pre-treated for 1 h, and subjected to additional studies. Cells were incubated for 24–72 h unless otherwise stated.

To analyze miR-140-5p expression in cardiac tissues, biopsy specimens were collected from intraventricular septum of IDCM patients*. *In Situ Hybridization (ISH) was performed using the IsHyb In Situ Hybridization Kit (BioChain, #K2191050) and miRCURY LNA miRNA ISH Buffer and Controls Kit (including scramble miRNA negative control probe and U6 snRNA positive control probe, # 339459) with a specific hybridization probe (LNA double-Digoxin (Dig)-labeled has-miR-140-5p probe, Qiagen #339111 YD00614358-BCG). The following steps were then performed on 5 μm-thick paraffin sections: following deparaffinization, samples were pre-digested with proteinase-K (10 μg/ml) for 10 min at 37 °C, pre-hybridized at 50 °C for 3 h, and slides incubated at 42 °C with denatured-probes (50 nM) in hybridization buffer for 16 h. Slides were then washed with saline-sodium citrate (SSC) buffer, and incubated with blocking solution for 1 h at room temperature. Slides were incubated with Alexa Fluor-647 IgG Fraction Monoclonal Mouse Anti-Dig (75 ng/ml, Jackson Immunoresearch, #200–602-156) for 2 h at room temperature. Hoechst 33,258 and Wheat Germ Agglutinin (WGA)-Alexa Fluor 488 Conjugate (Thermo Fisher Scientific, #H21491 and #W11261) were applied to detect nuclei or cell membrane. All slides were imaged by confocal microscope (Nicon, Japan).

### Staining of mitochondria with molecular probes

The NRVMs were placed into glass-bottomed dishes (Matsunami Glass, Japan), and 200 nM Mito Tracker Green FM (MitoGreen) (Life Technologies, M7514) and 200 nM Mito Tracker Red CM-H2Xros (MitoRed) (Life Technologies, M7513) were added to each well. The NRVMs were incubated for 45 min at room temperature in the dark. After staining, the cells were observed under a digital microscope (Keyence, Japan).

### Mice

All animal experiments were conducted in compliance with the protocol reviewed by the Institutional Animal Care and Use Committee of Niigata University and approved by the President of Niigata University. The study was carried out in compliance with the ARRIVE guidelines. The Mfn1^fl/fl^ mice were a kind gift from Professor Kenneth Walsh (Virginia University, USA). The MHC-Cre mice were purchased from Jackson Laboratories. The Mfn1^fl/fl^ mice and MHC-Cre mice were crossed to generate MHC-Cre; Mfn1^fl/fl^ mice, cardiac specific Mfn1 knockout mice (c-MfnKO). These mice underwent TAC as previously described^33^ at 10–11 weeks of age. The mice were analyzed two weeks after this operation. After the animals were euthanized by intraperitoneal (i.p) barbiturate injection, tissues were quickly collected for further analyses.

### Echocardiography

Ultrasonic echocardiography (UCG) was performed with a Vevo 2100 High Resolution Imaging System (Visual Sonics Inc.). To minimize variation of the data, cardiac function was only assessed when the heart rate was within the range of 550–650 beats/min. All studies for echocardiography were performed and analyzed in a blinded fashion in terms of genotypes.

### Metabolomic analyses

Metabolomic analyses were performed by Soga et al. using capillary electrophoresis/mass spectrometry (CE/TOFMS), as previously described^34^.

### Statistical analysis

Statistical analyses were performed with SPSS version 24 software. In some studies, outliers (shown as circles in figures) and/or abnormal values (shown as triangles in figures) were excluded by boxplot analyses for further analyses, and details are described in each figure legend. In some case, abnormal values were excluded from the description of the figure when they became out of range. Data are shown as the mean ± standard error of the mean (SEM). Differences between groups were examined by the two-tailed Student’s *t*-test or two-way analysis of variance (ANOVA), followed by Tukey’s multiple comparison test, the non-parametric Kruskal Wallis test, or the Dunnett’s test for comparisons among more than two groups. In all analyses, a *P* value of *P* < 0.05 was considered statistically significant.

## Supplementary Information

Supplementary Figures.

## Abbreviations

β-AR: Beta adrenergic receptor
IDCM: Idiopathic dilated cardiomyopathy
LVEF: Left ventricular ejection fraction
Mfn1: Mitofusin-1
NRVMS: Neonatal rat ventricular myocytes
TAC: Thoracic aortic constriction
