# Supplementary material for: Cardiac mitofusin-1 is reduced in non-responding patients with idiopathic dilated cardiomyopathy
Source: Sci Rep. 2021 Mar 24;11:6722. doi: 10.1038/s41598-021-86209-y (PMC7990924; doi:10.1038/s41598-021-86209-y)

Supplementary Information

Cardiac Mitofusin-1 is Reduced in Non-responding Patients with Idiopathic Dilated Cardiomyopathy

Yung Ting Hsiao[1, 2]*, Ippei Shimizu[1,3]*,**, Takayuki Wakasugi[1], Yohko Yoshida[1,3], Ryutaro Ikegami[1], Yuka Hayashi[1], Masayoshi Suda[1], Goro Katsuumi[1], Masaaki Nakao[1], Takuya Ozawa[1], Daisuke Izumi[1], Takeshi Kashimura[1], Kazuyuki Ozaki[1], Tomoyoshi Soga[4], Tohru Minamino[1, 2, 5]**

[1]*Department of Cardiovascular Biology and Medicine, Niigata University Graduate School of Medical and Dental Sciences, Niigata 951-8510, Japan*

[2]*Japan Agency for Medical Research and Development-Core Research for Evolutionary Medical Science and Technology (AMED-CREST), Japan Agency for Medical Research and Development, Tokyo, Japan.*

[3]*Division of Molecular Aging and Cell Biology, Niigata University Graduate School of Medical and Dental Sciences, Niigata 951-8510, Japan*

[4]*Institute for Advanced Biosciences, Keio University, Yamagata 997-0052, Japan*

[5]*Department of Cardiovascular Biology and Medicine, Juntendo University Graduate School of Medicine, Tokyo 113-8421, Japan*

Supplemental Figure 1

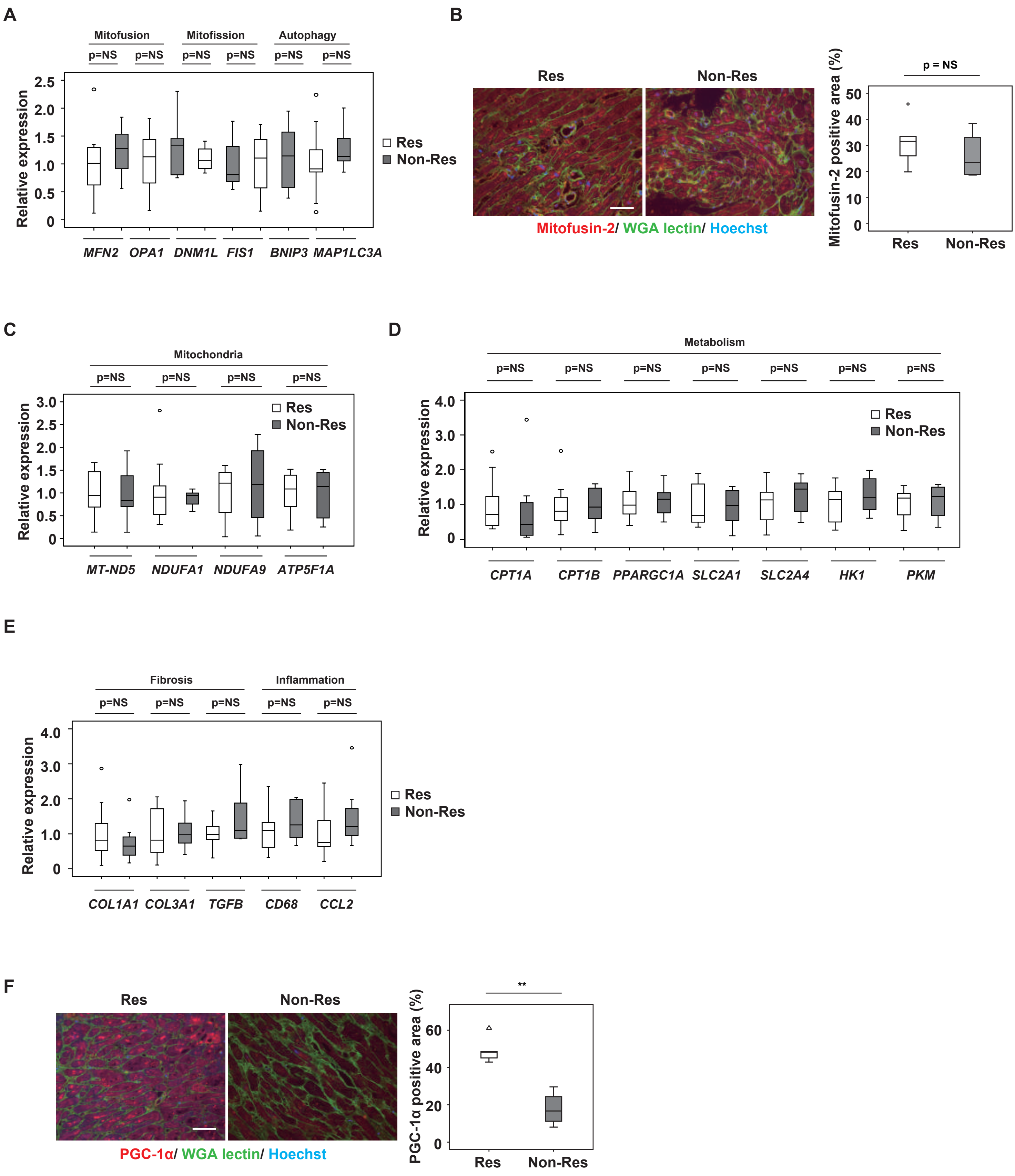

Supplemental Figure 1 Examinations of human samples

(A, C, D, E) Quantitative PCR of molecules related to mitofusion, mitofission, and autophagy (A), mitochondria (C), metabolism (D), fibrosis and inflammation (E) cardiac tissue of responders (Res) and non-responders (Non-Res). Outlier and abnormal values were excluded by boxplot (SPSS) for further statistical analysis. Some samples were not-detected, and also excluded from the analyses. The following numbers of samples for various molecules were analyzed: (*MFN2* (n=13, 8); *OPA1* (n=14, 8); *DNM1L* (n=4, 5); *FIS1* (n=14, 8); *BNIP3* (n=14, 8); *MAP1LC3A* (n=12, 7); *MT-ND5* (n=14, 8); *NDUFA1* (n=11, 6); *NDUFA9* (n=14, 8); *ATP5F1A* (n=14, 8); *CPT1A* (n=13, 7); *CPT1B* (n=13, 8); *PPARGC1A* (n=14, 8); *SLC2A1* (GLUT1) (n=14, 8); *SLC2A4* (GLUT4) (n=14, 8); *HK1* (n=14, 8); *PKM* (n=14, 8); *COL1A1* (n=13, 6); *COL3A1* (n=14, 8); *TGFB* (n=14, 8); *CD68* (n=12, 7), and *CCL2* (n=13, 6). (B, F) Immunofluorescence study for Mitofusin-2 (B) or PGC-1α (F). Right panels indicate positive area of respective molecules (B)(n=5,4)(F)(n=5,4). Scale bar=50μm. For study in Supplemental Figure 1B, an outlier n=1 in Res group, in Supplemental Figure 1F, an abnormal value n=1 in Res group were excluded from the analyses. Data were analyzed by two-tailed Student's t-test. *P<0.05, **P<0.01. Results are shown as mean ± SEM. NS = not significant. Small circle indicates outlier, triangle indicates abnormal value.

Supplemental Figure 2

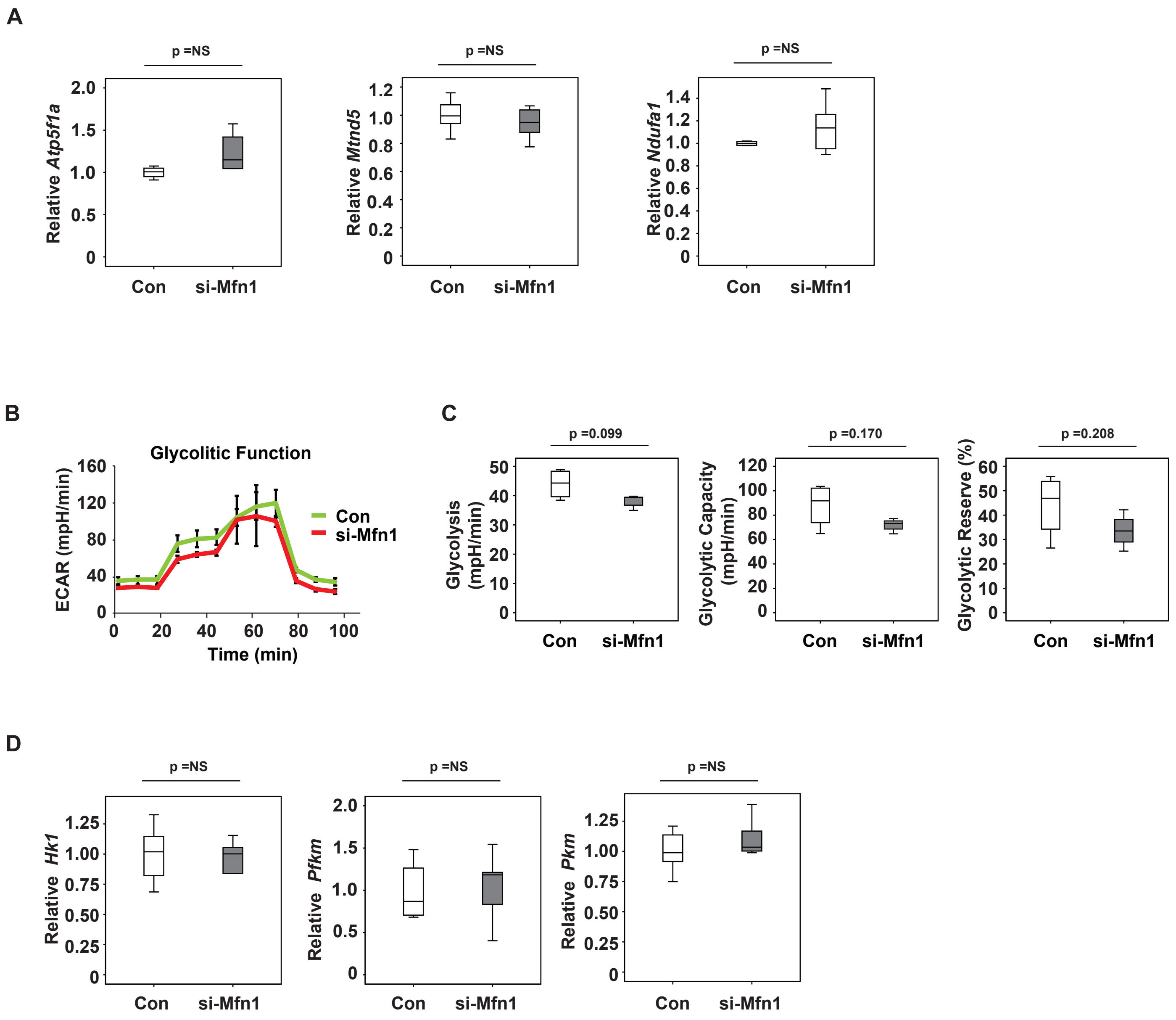

Supplemental Figure 2 Effects of si-Mfn1 in NRVMs

(A) Quantitative PCR of *Atp5fa1* (n=4, 6), *Mtnd5* (n=5, 6) and *Ndufa1*(n=4, 6) in NRVMs after introduction of control si-RNA (Con) or si-Mfn1. Abnormal values (n=1 in *Atp5fa1* (Con) and *Ndufa1* (Con)) were excluded by boxplot (SPSS) for further statistical analysis. (B, C) Evaluation of glycolysis with the Seahorse extracellular flux analyzer (B) and glycolysis (n=4, 5), glycolytic capacity (n=4, 4), and glycolytic reserve (n=4, 4) (C) in NRVMs after introduction of control si-RNA (Con) or si-Mfn1. Abnormal values (n=1 in glycolytic capacity (si-Mfn1) and glycolytic reserve (si-Mfn1)) were excluded by boxplot (SPSS) for further statistical analysis. (D) Quantitative PCR of *Hk1* (n=5, 6), *Pfkm* (n=5, 6) and *Pkm* (n=4, 6) in NRVMs after introduction of control si-RNA (Con) or si-Mfn1. An abnormal value (n=1 in *Pkm* (Con)) was excluded by boxplot (SPSS) for further statistical analysis. Data were analyzed by two-tailed Student's t-test. *P<0.05, **P<0.01. Results are shown as mean ± SEM. NS = not significant. Small circle indicates outlier, triangle indicates abnormal value.

Supplemental Figure 3

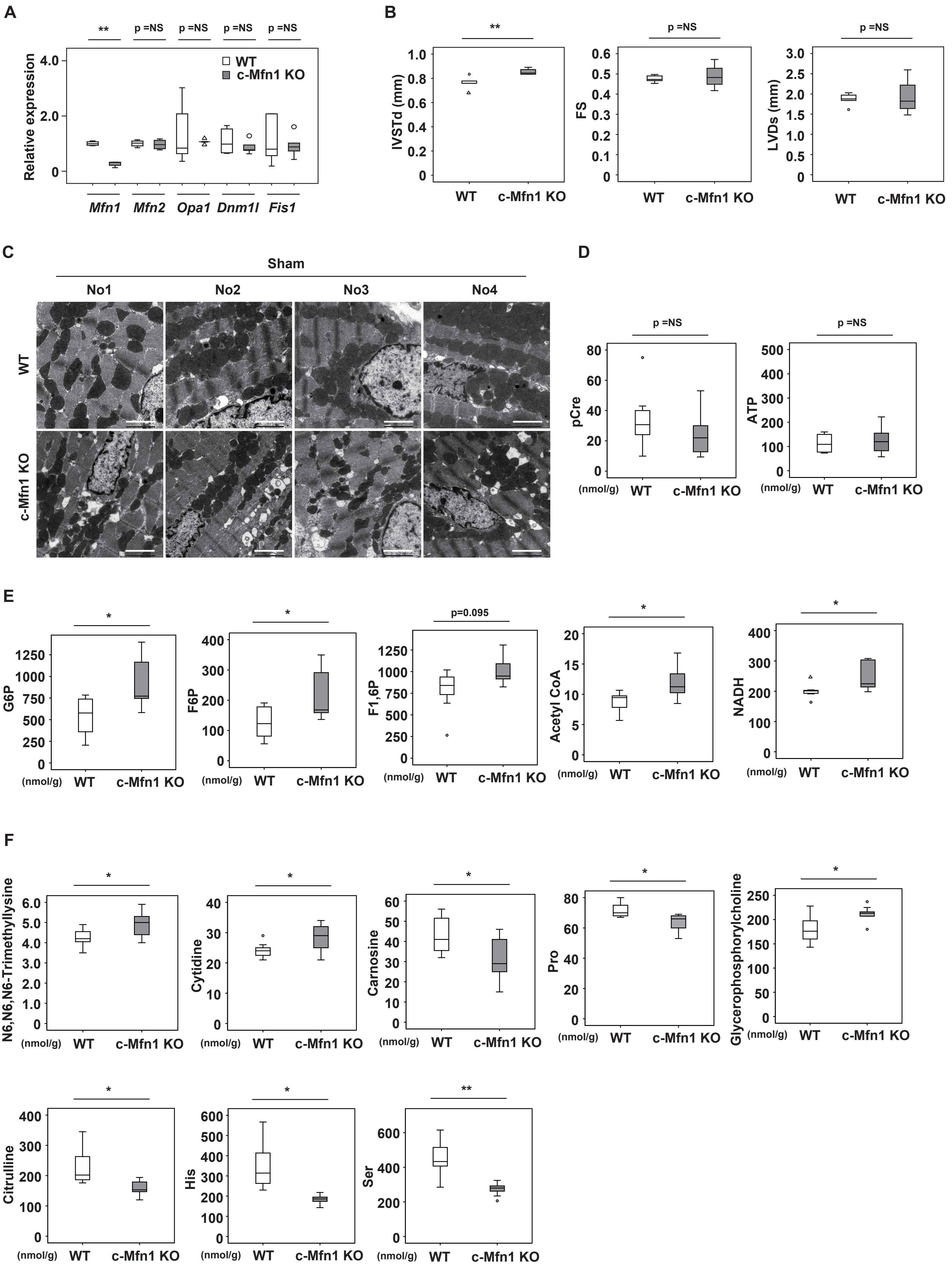

Supplemental Figure 3 PCR and metabolic analyses in c-Mfn KO mice

(A) Quantitative PCR of *Mfn1* (n=4, 4), *Mfn2* (n=4, 4), *Opa1* (n=4, 5), *Dnm1l* (n=4, 5), *Fis1* (n=4, 5) in cardiac tissues of wild type (WT) or c-Mfn1 KO mice. (B) UCG data of WT and c-Mfn1 KO mice at baseline. Interventricular septal thickness at end-diastole (IVSTd)(n=5, 4), Fractional shortening (FS) (n=5, 4) and left ventricular systolic dimension (LVDs) (n=5, 4) were analyzed. (C) Transmission EMs of cardiac tissues from WT and c-Mfn1 KO mice at baseline. No. C1-4 panels are from different mice. (D) Metabolomic study showing pCre or ATP levels in cardiac tissues of indicated mice at baseline (n=7, 9). (E, F) Metabolomic study showing anions (glucose 6-phosphate (G6P) (n=7, 9), fructose 6-phosphate (F6P) (n=7, 9), fructose 1,6-bisphosphate (F1,6P) (n=7, 9), acetylCoA (n=7, 9) and NADH (n=6, 9))(E), and cations N6,N6,N6-trimethyllysin (n=7, 9), cytidine (n=7, 9), carnosine (n = 7, 9), proline (Pro) (n=7, 9), glycerophosphorylcholine (n=7,8), citrulline (n=7,9), histidine (His) (n=7, 9) and serine (Ser) (n=7, 9))(F) of indicated mice. Abnormal values (n=1 in WT (NADH), n=1 in c-Mfn1 KO group (glycerophosphorylcholine)), and outliers (n=1 in WT F1,6P, n=2 in WT NADH, n=1 in WT cytidine, n=2 in c-Mfn1 KO glycerophosphorylcholine, n=1 in c-Mfn1 KO Ser) were excluded by boxplot (SPSS) for further statistical analysis. Data were analyzed by two-tailed Student's t-test. *P<0.05, **P<0.01. Results are shown as the mean ± SEM. NS = not significant. Small circle indicates outlier, triangle indicates abnormal value.

Supplemental Figure 4

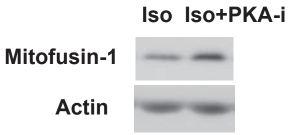

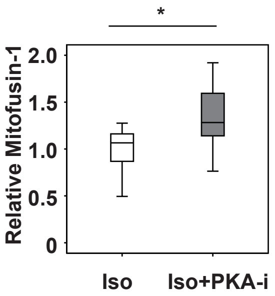

Supplemental Figure 4 Effects of isoproterenol on Mfn1 expression

Western blot analysis of Mfn1 in NRVMs administrated with isoproterenol (Iso) or isoproterenol+PKA inhibitor (Iso+PKA-i). Right panel indicates relative Mfn1 levels (n=11, 10). Data were analyzed by two-tailed Student's t-test. *$P<0.05$, **$P<0.01$. Results are shown as mean ± SEM. NS = not significant. Small circle indicates outlier, triangle indicates abnormal value.

Full blots

Figure 4

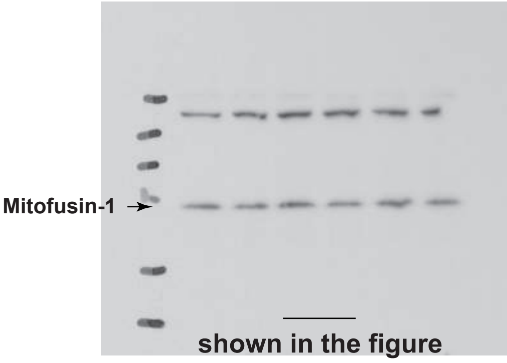

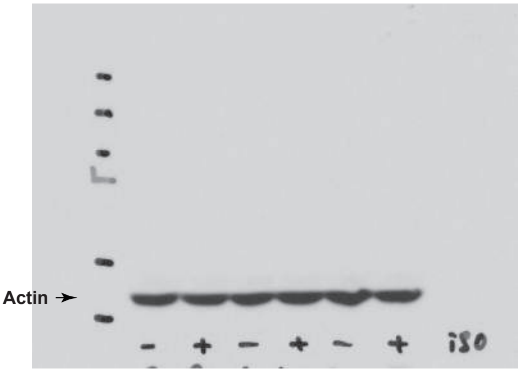

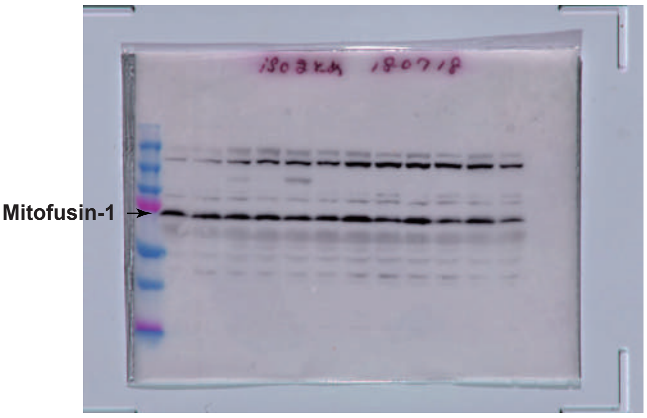

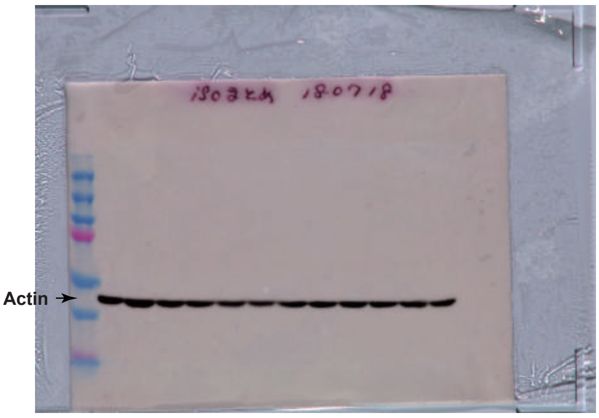

Supplementary Figure 4

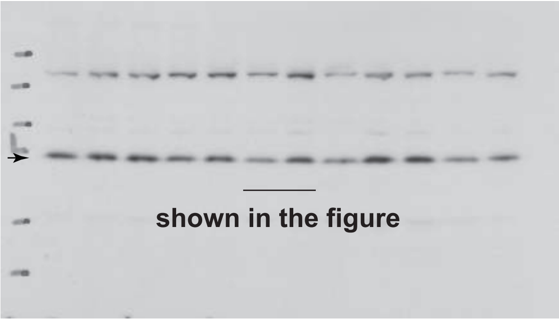

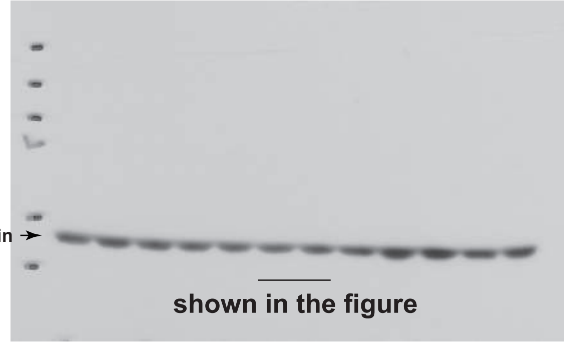

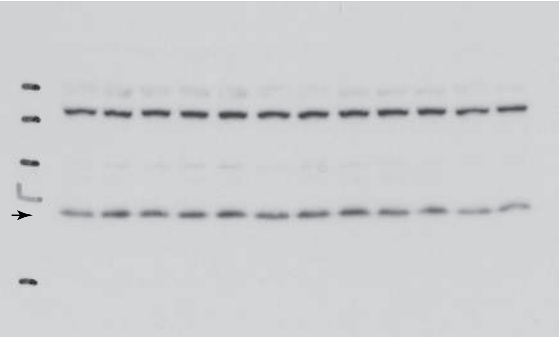

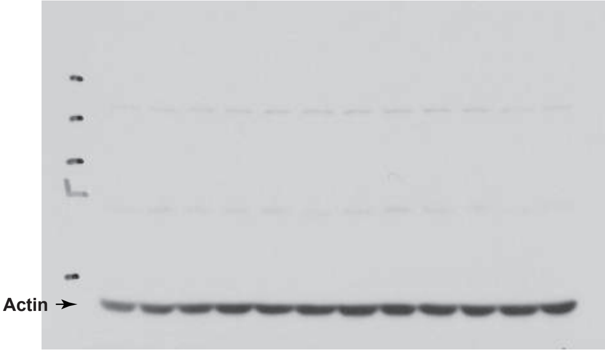

Supplement: Supplementary file 1 — Supplementary Figures. [file 41598_2021_86209_MOESM1_ESM.pdf]
